# Methanolic Bark Extract of *Abroma augusta* (L.) Induces Apoptosis in EAC Cells through Altered Expression of Apoptosis Regulatory Genes

**DOI:** 10.1155/2020/9145626

**Published:** 2020-04-13

**Authors:** Masum Miah, Ajmeri Sultana Shimu, Shafi Mahmud, Farjana-Binta Omar, Ratna Khatun, Sumon Chandro Mohanto, Kazi Md. Faisal Hoque, Md. Abu Reza

**Affiliations:** Molecular Biology and Protein Science Laboratory, Department of Genetic Engineering and Biotechnology, University of Rajshahi, Rajshahi 6205, Bangladesh

## Abstract

*Abroma augusta* (L.), one of the herbal medicinal plants, is widely used for treatment of various maladies. The present study was initiated to determine the antioxidant, hemolytic, cytotoxicity, and anticancer activities of methanolic extract from the bark of the plant. The phytochemical screening was done by analyzing different phytochemicals present in the extract. We observed the presence of alkaloids, steroids, terpenoids, flavonoids, reducing sugars, and glycosides in the bark extract which showed the highest antioxidant capacity. Antioxidant potential of the methanolic extract was evaluated *in vitro* by DPPH (2,2-diphenyl-1-picrylhydrazyl) scavenging assay method. This extract showed prominent scavenging activity with IC_50_ value of 38.65 *μ*g/ml. The hemolytic activity of the extract was evaluated at concentrations ranging from 250 to 1000 *μ*g/ml. It was observed that the extract induced hemolysis percentage of 9.41% to 4.1%, which implies that the extract has no potent hemolytic activity. Cytotoxicity and anticancer activities were observed on Ehrlich ascites carcinoma (EAC) cells. In addition, the bark showed promising cytotoxicity with IC_50_ value of 329.41 *μ*g/ml, and the study indicated that the extract was capable of inhibiting EAC cell growth by 75.5% when administered at 100 mg/kg/day body weight intraperitoneally for five consecutive days to Swiss albino mice. Morphological change of apoptotic cell was determined by fluorescence and optical microscopy. DNA fragmentation is another marker for apoptosis, and the bark extract-treated EAC cells showed smeared and fragmented DNA bands. Apoptosis correlated well with the upregulation of p53 and Bax and also with the downregulation of NF-*κ*B and Bcl-2. Furthermore, activity and interaction of two *A. augusta* compounds were tested through molecular docking simulation study. In conclusion, our results suggest that *A. augusta* bark has the potential to be considered as an anticancer agent.

## 1. Introduction

Cancer is an attenuating disease that is known as one of the major health problems of global concern and accounts for an estimated 9.6 million deaths in 2018 [1, 2]. Generally, cancer refers to any one of a large number of diseases characterized by abnormal cell growth with the potential to invade other normal cells of the body. The current treatment options for cancer include chemotherapy, radiotherapy, and chemically derived drugs; those are generally nonspecific, cause toxicity, damage normal cell, and even lead to death [3]. According to the World Health Organization, about 65% of the global population tend to choose traditional herbal medicines to treat various diseases like cancer, diabetes, and hepatic disorder because of their availability, absence of adverse effects, and cost effectiveness [4]. Scientists are constantly in search of phytochemicals from medicinal plants with unique features to develop novel drugs against cancer in contrast to conventional chemotherapy, radiotherapy, and surgery to diminish adverse side effects [5, 6]. Earlier reports show that some plant-derived natural phytochemicals have promising anticancer potential and ability to reduce the growth of specific tumor cells [7].

Apoptosis plays vital roles in a broad sense of physiological processes during fetal development as well as in adult tissues. Apoptosis takes place through differential regulation of various types of proapoptotic and antiapoptotic genes [8]. Stirring of cell morphology via chromatin condensation and nuclear fragmentation, plasma membrane blebbing, and cell shrinkage are the hallmarks of apoptosis [9]. During cancer formation, expression of antiapoptotic genes is upregulated, whereas proapoptotic genes are downregulated and thus cells escape apoptosis to become cancerous. Proapoptotic p53, Bax signaling and antiapoptotic Bcl-2, NF-*κ*B signaling play critical roles in tumor development and progression and are involved in angiogenesis, metastasis, and cell survival [10, 11].

Antioxidants are substances that scavenge free radicals and protect oxidative damage by neutralizing the reactive oxygen species (ROS) [12]. Overproduction of ROS contributes to oxidative stress in the human body, which leads to damage of proteins, lipids, and DNA that is associated with chronic degenerative diseases such as hypertension, coronary artery diseases, diabetes, and cancer [13]. Photochemical derived from plants contain phenolics, flavonoids, alkaloids, and tannins, which may possess high antioxidant activity and also may protect the body from several degenerative disorders [14].

*A. augusta* (L.) (family: Sterculiaceae or Malvaceae in some classifications) is commonly known as Ulatkambal in Bengali and Hindi, and Devil's cotton in English. It is an evergreen small shrub with a long history of medicinal uses and mostly found in tropical Asia, South Eastern Africa, and Australia [15]. In India, the mother tincture of *A. augusta* is widely used in homeopathic medicine to treat uterine disorders and diabetes mellitus [16, 17]. Its root-bark is highly useful as uterine tonic and is used to treat amenorrhoea and dysmenorrhea, with abortifacient and antifertility activities, whereas leaves are used for diabetes, rheumatic pains, gonorrhea, headache, and sinusitis [18]. However, the anticancer potential of this medicinal plant is still unrevealed and no advanced molecular study of gene-mediated oncogenic pathway was conducted so far. Therefore, the current study aims to investigate the anticancer potential of *A. augusta* and the molecular pathway through which it induces apoptosis of cancer cells. The study is again strongly supported by molecular docking study of *A. augusta* derived molecules interacting with Bcl-2 and NF-*κ*B gene.

## 2. Materials and Methods

### 2.1. Collection of Plants Material

The bark of the *A. augusta* was collected from the Shah Sufi Mosque, Chhatak, Sylhet, Bangladesh, during July 2017 (latitude: 25°02′ 30.12″ N, longitude: 91°40′ 30.00″ E). The identity of the plant was authenticated by Professor Dr. AHM Mahbubur Rahman, taxonomist in the Department of Botany, University of Rajshahi, Bangladesh, where a voucher specimen (Accession number: 17) was deposited.

### 2.2. Chemicals and Reagents

Methanol, 2,2-diphenyl-1-picrylhydrazyl (DPPH), potassium ferricyanide, phosphate buffer, catechin (CA), ferrous ammonium sulphate, butylated hydroxytoluene (BHT), gallic acid (GA), and FeCl_3_ were purchased from Sigma Aldrich Chemical Co. (St. Louis, MO, USA); Folin–Ciocalteu phenol reagent (FCR), trypan blue dye, DAPI, DMSO, and sodium carbonate were obtained from Merck (Darmstadt, Germany); 2′,7′-dichlorofluorescein diacetate was acquired from Sigma Aldrich (USA). RNAsimple total RNA kit, M-MLV reverse transcriptase, dNTPs, and oligo (dT) primers were purchased from TIANGEN Biotech (Beijing, China); primers for GAPDH, p53, Bax, Bcl-2, and NF-*κ*B were custom-synthesized from IDT, Malaysia.

### 2.3. Preparation of Plant Extract

*A. augusta* bark sample was thoroughly washed with distilled water and shade-dried for 7 days with occasional sun drying. The dried materials were ground into fine powder using a mechanical grinder and stored at room temperature (RT) for future use. 100 gm of bark powder was immersed in 500 ml methanol and shaked at 200 rpm for 24 hours by shaking incubator (HYSC, Hanyang Scientific Equipment Co., Ltd., Seoul, Korea). Then, the solution was filtered through Whatman No. 1 filter papers and concentrated with a rotary evaporator. Finally, the whole solvent was evaporated using freeze dryer. The extract was then kept in glass vial with airtight caps and stored at 4°C.

### 2.4. Qualitative Phytochemical Screening

The methanolic extract was used to perform the phytochemical screening according to the methods described by Sofowora and Trease and Evans [19, 20].

### 2.5. Determination of Total Phenolics

Total phenolic contents of the extracts were determined by Folin–Ciocalteu method described by Machu et al. [21]. In brief, an aliquot of the extracts, 300 *μ*l (1 mg/ml DW), was mixed with 2.25 ml Folin–Ciocalteu reagent (previously diluted with water 1 : 10 v/v) and 2.25 mL (75 g/L) of sodium carbonate. The tubes were vortexed thoroughly for 15 s and allowed to stand for 90 min at 25°C for color development. Absorbance was then measured at 725 nm in a UV-Vis spectrophotometer (GENESYS 10S, Thermo Scientific, USA). Samples of extracts/standard were evaluated at a final concentration of 1 mg/ml. Total phenolic contents were expressed in terms of gallic acid equivalent (GAE) (standard curve equation: *y* = 0.003*x* − 0.002, *R*^2^ = 0.990), mg of GAE/g of dry extract.

### 2.6. Determination of Total Flavonoids

Total flavonoid contents were estimated using aluminum chloride colorimetric assay method described by Kiranmai et al. [22]. 150 *μ*l of 5% sodium nitrate and 2.5 mL of distilled water were added to 0.5 ml of crude extract (1 mg/ml DW) into a microcentrifuge tube. After 6 min, 0.3 ml of 10% AlCl_3_ was added and the mixture was allowed to stand for 5 min followed by addition of 1 mol/l NaOH solution. The mixture was thoroughly mixed using vortex and the absorbance of the mixtures was measured at 510 nm. Total flavonoid contents were expressed in terms of catechin equivalent (CAE) (standard curve equation: *y* = 0.004*x* − 0.054, *R*^2^ = 0.990), mg of CAE/g of dry extract.

### 2.7. DPPH Free Radical Scavenging Assay

Free radical scavenging ability of the extract was determined according to Matkowski and Piotrowska [23]. 10 mg DPPH was dissolved in 100 ml methanol (MeOH) to obtain a concentration of 100 *μ*g/ml. The stock solution of the extracts was prepared by dissolving 1 mg freeze-dried extract in 1 ml MeOH. Dilutions of the stock solutions were prepared to obtain concentrations of 25 *μ*g/ml, 50 *μ*g/ml, 100 *μ*g/ml, 150 *μ*g/ml, and 200 *μ*g/ml, while the control was prepared by dissolving 1 ml DPPH in 1 ml MeOH (without sample). 1.5 ml methanol solution of DPPH was added to each solution, and the solution was kept in dark for 30 min. The absorbance of the solution was measured spectrophotometrically at 517 nm, and BHT was used as standard. The percentage of DPPH radical scavenging activity was calculated by the following equation:

(1) $$\begin{array}{c} \%\text{ DPPH radical scavenging activity}=\frac{\left(A_{0}-A_{1}\right)}{A_{0}}\times 100, \end{array}$$

where *A*_0_ is the absorbance of the control and *A*_1_ is the absorbance of the extracts. Then, the percentage of inhibition was plotted against concentration, and from the graph IC_50_ was calculated.

### 2.8. Experimental Animals

A total of 24 mature female Swiss albino mice (25–30 g) were collected from the Department of Pharmacy of Jahangirnagar University, Dhaka, Bangladesh. These animals were kept in standard laboratory conditions (temperature 25 ± 2°C; humidity 55 ± 5%) with 12 h dark/light cycle throughout the whole study. To keep the hydration rate constant, food and water supply were stopped 12 hours before the experiment.

### 2.9. Ethical Approval

The current experimental procedure was approved by the Institutional Animal, Medical Ethics, Biosafety and Biosecurity Committee (IAMEBBC) for Experimentations on Animal, Human, Microbes and Living Natural Sources, Institute of Biological Sciences, University of Rajshahi, Bangladesh (Approval no.: 225/320-IAMEBBC/IBSc).

### 2.10. Transplantation of Tumor

Ehrlich ascites carcinoma (EAC) cells were obtained from Indian Institute of Chemical Biology, Kolkata, India. The EAC cells culture and aspiration were maintained according to method of Alam [24]. In brief, *in vivo* cultured EAC cells were drawn out from 6- to 7-day-old ascites tumors of EAC tumor-bearing mice. The drawn cells were diluted with 0.9% normal saline. After dilution, the numbers of EAC cells were adjusted to approximately 1 × 10^6^ cells/ml by counting the cell number with the help of a hemocytometer. Each animal received 0.1 ml of tumor cell suspension containing 1 × 10^6^ tumor cells intraperitoneally.

### 2.11. Acute Toxicity Study

The acute toxicity was studied by administering 1000 mg/kg b. wt oral dose of the *A. augusta* bark extract as described earlier [25]. No mortality was observed during the study. On the basis of this study, the doses of 50 and 100 mg/kg b. wt/day (i.p.) were selected for *A. augusta.*

### 2.12. Hemolytic Activity

Hemolytic activity of methanolic bark extract of *A. augusta* was assayed on goat erythrocytes following the method of Henkelman et al. [26]. In brief, 5 ml of blood was obtained from healthy goat in tubes containing 3.8% trisodium citrate and centrifuged at 5,000 rpm for 20 min. The supernatant was discarded by micropipette, and afterwards the blood cells were washed two times with 0.9% NaCl at 5,000 rpm for 15 min to prepare 10% RBC. 150 *μ*l of 10% RBC suspension was mixed with 25, 50, and 100 *μ*l of test samples (10 mg/ml). Two controls were prepared without extract; negative control received 1350 *μ*l of 0.9% NaCl, while positive control received 1350 *μ*l of dH_2_O; reaction mixture was incubated at 37°C in a water bath for 60 min. The volume of the reaction mixture was diluted to 1.5 ml by adding 0.9% NaCl. Finally, it was centrifuged at 3000 rpm for 5 min, and the resultant hemoglobin concentration in the supernatant was spectrophotometrically measured at 540 nm. The percentage of hemolysis was calculated by dividing sample absorbance on positive control absorbance (complete hemolysis) multiplied by 100.

### 2.13. MTT Assay

Cytotoxic activity of bark extract of *A. augusta* was evaluated in 96-well microtiter plates against EAC cell by MTT (3-[4, 5-dimethylthiazol-2-yl]-2,5-diphenyl tetrazolium bromide) colorimetric assay [27]. For experiment, 200 *μ*l of EAC cells (1 × 10^6^ cells) cultured in RPMI-1640 media was taken in each well of a 96-well culture plate at different concentrations (500, 250, 125, 62.5, and 31.25 *μ*g/ml) of extract. Culture plate was then placed in CO_2_ incubator at 37°C with constant flow of 5% CO_2_ for 1 day followed by the removal of supernatant. After addition of 180 *μ*l of PBS and 20 *μ*l of MTT to each well of the culture plate, they were incubated at 37°C for an additional 8 hours. The supernatant was removed again and 200 *μ*l of acidic isopropanol was put into each well followed by incubation at 37°C for 1 hour. The plate was read at 570 nm on a multiwell plate reader (Multiskan™ FC Microplate Photometer, Thermo Scientific, Waltham, MA, USA). The following equation was used to calculate the cell proliferation inhibition ratio:

(2) $$\begin{array}{c} \text{proliferation inhibition ratio}\left(\%\right)=\left[\frac{\left(A-B\right)}{A}\right]\times 100, \end{array}$$

where *A* and *B* denote the absorbance OD_570_ nm of the cellular homogenate in the absence and presence of the bark extract, respectively.

### 2.14. Determination of Cell Growth Inhibition (*In Vivo*)

To evaluate the cell growth inhibition [28] of bark extract of *A. augusta*, four groups of Swiss albino mice (*n* = 6) were used. For therapeutic evaluation, 1 × 10^6^ EAC cells were inoculated in every mouse on day 0. Treatments were started after 24 h of tumor inoculation and continued for 5 days. Group one was used as control. Group two received bleomycin (standard anticancer drug) at the dose of 0.3 mg/kg/day. Group three received the bark extract at the dose of 50 mg/kg (i.p.). Group four received the bark extract at the dose of 100 mg/kg (i.p.). Mice in each group were sacrificed on day six and total intraperitoneal tumor cells were harvested by normal saline (0.98%). Viable cells of EAC cells of both treated and control mice were first identified by using trypan blue and then counted by a hemocytometer. The cell growth inhibition was calculated using the following formula:

(3) $$\begin{array}{c} \%\text{ cell growth inhibition}=\left(\frac{1-T_{w}}{C_{w}}\right)\times 100, \end{array}$$

where *T*_*w*_ is the mean number of EAC cells in treated mice and *C*_*w*_ is the mean number of EAC cells in control mice.

### 2.15. DAPI Staining for Apoptosis Assessment

EAC cells were collected from both treated and control mice after 5 consecutive days of treatment [29]. DAPI (0.1 *μ*g/ml) was used for staining the cells at 37°C for 15 min in dark condition. Washing and resuspension were carried out by PBS in order to find out morphological alterations. Moreover, 5 *μ*l supernatant was taken on a microscopic slide and cellular changes were observed under the fluorescence microscope (Olympus IX71, Korea).

### 2.16. DNA Fragmentation Assay

The effect of the extract on DNA fragmentation was determined by previously described method [30]. After 5 consecutive days of treatment, both treated and control EAC cells were collected. Genomic DNA was isolated from the cells by using TIANamp Genomic DNA kit (TIANGEN Biotech, Beijing, China), and the DNA was analyzed on 1% agarose gel under gel documentation system (Alphaimager Mini System, ProteinSimple).

### 2.17. Determination of Intracellular ROS Generation

By using 2,′7′-dichlorodihydrofluorescein diacetate (DCF-DA), intracellular reactive oxygen species (ROS) levels of living cells were measured as described previously [31]. The EAC cells-bearing mice were treated with bark extract of *A. augusta*, and on the 6^th^ day mice were sacrificed and tumor cells were collected. Treated and untreated cells were incubated with DCF-DA (50 *μ*M) as the fluorescence agent for 30 min at 37°C and subsequently washed with PBS. The fluorescence was monitored by an inverted fluorescent microscope (Olympus IX71, Korea) at an excitation and emission wave length of 485 nm and 530 nm, respectively.

### 2.18. RNA Isolation, cDNA Synthesis, and PCR Amplification of p53, Bax, Bcl-2, and NF-*κ*B

Total RNA was extracted from EAC cells collected from mice of untreated and treated (100 mg/kg/day) groups on the sixth day after EAC cell implantation using TIANGEN reagent kit. RNA was quantified using a nanodrop (Thermo Scientific, Waltham, Massachusetts, USA). RNA was also run on a formamide gel to check the quality in visual way. cDNA synthesis was carried out using reverse transcription reaction mixture (20 *μ*l), containing 2 *μ*l oligo (dT), 1 *μ*l M-MLV reverse transcriptase, 2 *μ*l dNTPs, 4 *μ*l 5× 1^st^ strand buffer, and 3 *μ*l total RNA sample; 8 *μ*l dH_2_O. 2 *μ*g of total RNA was reverse-transcribed into cDNA in a 20 *μ*l reaction mixture containing 2 *μ*l oligo (dT), 1 *μ*l M-MLV reverse transcriptase, 2 *μ*l dNTPs, 4 *μ*l 5× 1^st^ strand buffer, and required amount of deionized water. Relative expression of the cancer genes was investigated using the primer pairs in Table 1. The primers (IDT, Singapore) were designed based on previous report [24].

### 2.19. Brine Shrimp Lethality Bioassay

Cytotoxicity of the bark extract of *A. augusta* was screened against *Artemia salina* according to published protocol [34]. For the experiment, 1 mg of the bark extract was dissolved in 1 ml (1000 *μ*l) of distilled water to get a concentration of 1 *μ*g/*μ*l, in addition to serial dilution. Various concentrations of bark extract such as 25, 50, 75, 100, 150, and 200 *μ*g/mL were obtained. After 24 h of incubation, the percentage of mortality of the nauplii was calculated for each concentration and the LD_50_ value was determined by using probit analysis.

### 2.20. Ligand Preparation

Reported structures of photochemical compounds from *A. augusta* (CID-72, CID-247, CID-305, CID-8468, CID-73170, CID-73659) were retrieved from PubChem database as SDF format [33]. To remove clashes among the atoms of the ligands, energy minimization was carried out using YASARA server by utilizing YASARA software [34].

### 2.21. Protein Preparation

Crystal structure of Bcl-2 and NF-*κ*B protein was retrieved from Protein Data Bank database [35] with their respective PDB ID (PDB-2W3L and 1SVC). Energy minimization of the protein was done through Swiss-PdbViewer software [36]. Improper bonding, missing hydrogen, and side chain geometry were checked. PyMOL software package [37] was used to remove water molecules and hetero atoms. Finally, protein and ligand were saved as PDBQT format which is the only supported file in AutoDock Vina [38].

### 2.22. Pharmacokinetic Parameter

admetSAR [39] and SwissADME [40] web servers were utilized to access the information of compound metabolism, carcinogenicity, and Lipinski filtering. Structure-data file (SDF) and simplified molecular-input line-entry system (SMILES) were used as input system to unlock pharmacological properties of the ligand.

### 2.23. Docking

After ADMET screening, compounds were subjected to molecular docking in AutoDock Vina. Crystal structures of Bcl-2 and NF-*κ*B were used as micromolecules for docking study. Energy minimization was done by universal force field (UFF) and conjugated gradient algorithm. The total number of steps and the number of steps for update were set as defaults [41]. In molecular docking process, the center grid box was set at *X*: 42.336, *Y*: 26.3284, *Z*: 35.9666 and box size was *X*: 827210, *Y*: 70.1628, *Z*: 64.1580 for NF-*κ*B protein (Table 2). On the other hand, for Bcl-2 protein, center grid box was set at *X*; 47.3586, *Y*: 33.2146, *Z*: −5.4377 and box size was *X*: 50.00, *Y*: 45.64, *Z*: 42.3887 (Table 3). In addition, known inhibitors bleomycin was used in this study as control in docking study. This FDA approved drug was prepared and minimized by using previous method as described in ligand preparation section. MOE dock module of MOE-2015 software was used to cross-validate the docking score obtained from AutoDock Vina. Alpha Matcher placement and Alpha Triangle placement method were applied to dock into the active site of the protein. GBVI/WSA dG with the force field refinement was used to predict binding affinity. Hydrophobic interaction, hydrogen bonding which was responsible for the protein and ligand interaction, and these interactions were assessed through Discovery Studio [42] and PyMOL [43].

### 2.24. Statistical Analysis

Data were represented as mean ± standard deviation (SD) for triplicate experiments. Statistical analysis was performed by one-way ANOVA, and *p* < 0.05 was considered as statistically significant. SPSS 16.0 software for Windows 10 was used for statistical analysis.

## 3. Results

### 3.1. Phytochemical Screening

The phytochemical screening test of *A. augusta* bark extract exhibited the presence of alkaloids, glycosides, reducing sugars, terpenoids, steroids, and flavonoids (Table 4), which is indicated by a positive sign (+), while the absence of saponins and tannins is indicated by a negative sign (−).

### 3.2. Total Phenolic and Flavonoid Contents

Polyphenols are important compounds of plant due to their antioxidant nature and various diseases curing abilities. Therefore, the amount of total phenolic compounds and flavonoids from *A. augusta* bark was quantified as shown in Table 5.

### 3.3. DPPH Radical Scavenging Activity

**Figure 1:**
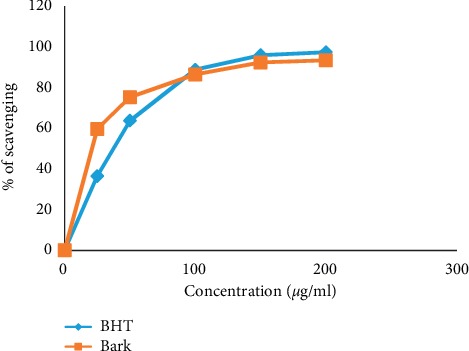
The antioxidant activity of bark extracts of *A. augusta* along with standard BHT. The scavenging percentage of DPPH by *A. augusta* at the concentration rate (25–200 *μ*g/ml) as compared to BHT.

Figure 1 shows free radical scavenging activity of *A. augusta* bark extract and standard BHT. The current study shows that the bark extract and standard BHT demonstrate almost equal amount of activity. The scavenging activity of bark extract at the highest concentration of 200 *μ*g/ml was 93.32%, whereas at the same concentration the activity of standard was 97.31%. The IC_50_ value of bark extract and BHT standard was calculated to be 38.65 *μ*g/ml and 57.29* μ*g/ml, respectively. A higher IC_50_ value means lower radical scavenging activity. Hence, the free radical scavenging activity of bark extract is greater than that of BHT.

### 3.4. Hemolytic Activity

Human erythrocytes membranes from blood types show different stability as determined from the mean corpuscular fragility. Chemical agent can positively affect the red cell membrane, and on the contrary, many of them have severe undesirable effects, which induce hemolytic anemia. As bark extract is likely to be used as drug, it needs to be studied for its potential hemolytic activity. The hemolytic activity of the extract was evaluated at concentrations of 250 *μ*g/ml, 500 *μ*g/ml, and 1000 *μ*g/ml (Table 6). It was observed that the extract induced nonsignificant amount of hemolysis, which implies that the extract has no potent hemolytic activity.

### 3.5. MTT Assay

**Figure 2:**
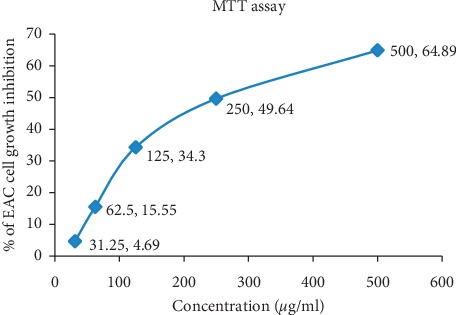
*In vitro* cell growth inhibition along with IC_50_ value of bark extract of *A. augusta* by MTT colorimetric assay. EAC cells were treated at different doses of bark extract for 24 h in RPMI-1640 medium to evaluate the EAC cells growth inhibition, and IC_50_ values were calculated from the dose-response curve.

The study of cytotoxicity of bark extract against EAC cells is presented in Figure 2, which revealed that the *A. augusta* bark extract showed promising cytotoxic activity against cancer cell. 500 *μ*g/ml, 250 *μ*g/ml, 125 *μ*g/ml, 62.5 *μ*g/ml, and 31.25 *μ*g/ml concentration of bark extract induced 64.89%, 49.64%, 34.30%, 15.55%, and 4.69% cell death, respectively, against EAC cells. The IC_50_ value of bark extract of *A. augusta* was calculated as 329.41 *μ*g/ml.

### 3.6. Growth Inhibitory Activity of Bark Extract of *A. augusta* against EAC Cells

**Figure 3:**
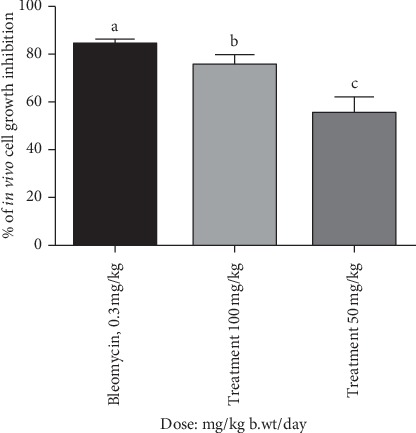
*In vivo* comparison of cell growth inhibition of EAC-bearing mice treated with different doses of bark extract of *A. augusta*. Significant cell growth inhibition was observed in EAC cells in response to 50 mg/kg/day and 100 mg/kg/day of *A. augusta* extract when compared with standard (bleomycin). Data were expressed in mean ± SD (Table 6) at ^*∗*^*p* < 0.05 (*n* = 6) significant level.

The antiproliferative activity of bark extract of *A. augusta* was observed using hemocytometer by counting EAC cells using trypan blue dye. Results show that the viability of EAC cells is considerably decreased in treated group in comparison with control (Table 7; Figure 3). Maximum cell growth inhibition (75.5% reduction of tumor cells) was found with the treatment of bark extract at the dose of 100 mg/kg body weight (i.p.).

### 3.7. Detection of Apoptotic EAC Cells by DAPI Staining

**Figure 4:**
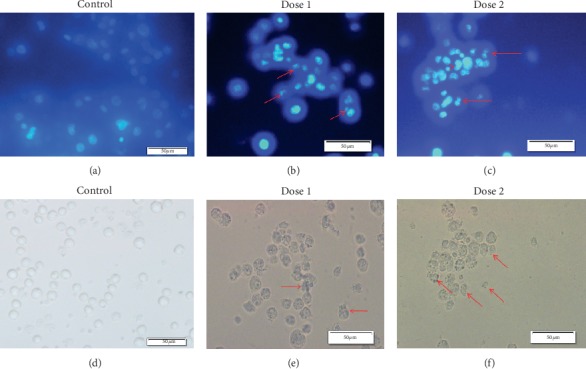
Fluorescence and optical detection of apoptosis using DAPI staining in untreated and treated EAC cells. Regular uniform shape of the cells was found in control in (a) fluorescence microscopy and (b) optical microscope. EAC cells treated with bark extract (100 mg/kg) of *A. augusta* and stained with DAPI in (c) fluorescence microscopy and (d) optical microscope showed apoptotic features indicated by arrows. At 200 mg/kg bark extract-treated EAC cells revealed similar changes in (e) fluorescence microscopy and (f) optical microscope.

To observe the morphological changes of EAC cells after five consecutive days of treatment, DAPI staining assay was performed. Treated cells showed different hallmarks of apoptosis which include shrunken nature of cell, condensation of chromatin, membrane blebbing, and nuclear fragmentation of apoptotic bodies when compared to the normal cells (Figure 4).

### 3.8. New Measurement of ROS Generation in EAC Cells

**Figure 5:**
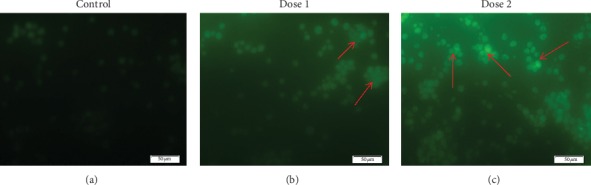
ROS generation in EAC cells treated with bark extract of *A. augusta*. (a) The dark staining of nuclei means that the intracellular generation of ROS was found in control. (b) A large number of EAC cells showed light staining for ROS (arrows) after treating EAC cells with 50 mg/kg bark extract of *A. augusta*. (c) An intense staining formation of ROS observed in EAC cells treated with 100 mg/kg bark extract of *A. augusta*.

The ROS production in EAC cells was determined after 5 consecutive days of treatment. As shown in Figure 5, control cells showed faintly green fluorescence that indicates low level of ROS production showing that the ROS formation was of low level. The methanolic bark extract increased ROS generation in EAC cells in a concentration dependent manner. The ROS generation was increasing at higher concentration (50 and 100 mg/kg/day). In EAC cells, the highest ROS generation was observed at 100 mg/kg/day of bark extract of *A. augusta*. The result demonstrated that the *A. augusta* bark extract induced mitochondria-mediated apoptosis in EAC cells which may be related to ROS hypergeneration.

### 3.9. DNA Fragmentation Assay

**Figure 6:**
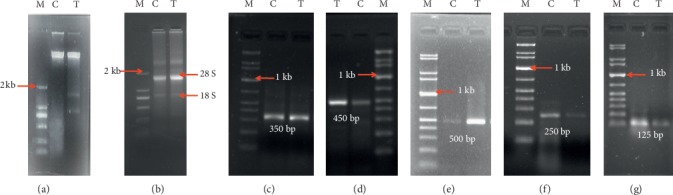
Different expression of proapoptotic and antiapoptotic genes by semiquantitative RT-PCR. Lanes (M), (C), and (T) represent molecular marker, control, and bark extract-treated cells, respectively. In (a) and (b), DL2000 DNA marker (Hubein Aibo Technology Co. Ltd.) was used, and in (c)–(g), 1 kb plus DNA ladder (TIANGEN Biotech Co., Ltd.) was used. (a) Fragmentation of genomic DNA in EAC cells treated with 100 mg/kg b.wt of *A*. *augusta* bark extract for 5 consecutive days. (b) Isolated RNA exhibited two separate bands as 28S and 18S. (c) GAPDH (housekeeping gene) expression as a control. (d, e) The upregulation of the proapoptotic genes p53 and Bax compared to control. (f, g) The downregulation of the antiapoptotic genes Bcl-2 and NF-*κ*B in case of treated cells compared to control.

The DNA fragmentation by agarose gel electrophoresis pattern of DNA from normal and bark extract-treated EAC cells is presented in Figure 6(a). In control mice, there was no fragmented band, whereas smeared and fragmented DNA bands were found in case of bark extract-treated EAC cells, shown in Figure 6(a). This may be due to chromatin condensation and nuclear fragmentation.

### 3.10. Modulation of Apoptotic Gene

RT-PCR analysis of the mRNA levels of p53, Bax (proapoptotic) and Bcl-2, NF- *κ*B (antiapoptotic) was performed to evaluate the expression of apoptosis genes (Figure 6). GAPDH (housekeeping gene) was used as control (Figure 6(c)). The figures represented the changes of apoptotic genes in treated and untreated cells. p53 is a tumor suppressor gene that is overexpressed in bark extract-treated cells at the 450 bp position on the gel; also, the expression of Bax (proapoptotic) gene is increased by treatment with bark extract at the 500 bp position on the gel. Bcl-2 gene expression in cells treated with bark extract was reduced compared to control at 250 bp position on the gel which was shown in Figure 6(f). Similarly, NF-*κB* was overexpressed in control at 125 bp position on the gel, whereas it was downregulated in bark extract-treated cells.

### 3.11. Brine Shrimp Lethality Bioassay

The LD_50_ value of bark extract was calculated using probit analysis. Results of the lethality bioassay have been tabulated in Supplementary Table ([Supplementary-material supplementary-material-1]). The extract showed LD_50_ 391.539 *μ*g/ml in probit analysis, which is shown as Supplementary Figure 1 ([Supplementary-material supplementary-material-1]). The lower LD_50_ means higher toxicity. The result proves that bark extract of *A. augusta* exhibits low toxic effects against brine shrimp nauplii in comparison to EAC cells.

### 3.12. ADMET

Physiochemical characteristics of a compound can be valid alternative to experimental method. In order to propose a compound to be drug, it has to maintain certain criteria. Here we predicted pharmacological and pharmacokinetics properties of the chemical compound of *A. augusta*. Therefore, admetSAR and SwissADME web servers were used to determine whether the ligand has any toxic profile by evaluating the physiological profile. This prediction method involves blood-brain barrier, human intestinal absorption, P-gp inhibitor, subcellular localization, hERG profile, AMES toxicity, carcinogen, and Lipinski's rule of five of the selected compound. ADMET filtering showed that four chemical compounds of *A. augusta* had carcinogenic effect and did not follow Lipinski's rule of five (H-bond donors < 5, H-bond acceptors < 10, MW<500 dalton, C log *p* > 5, rotatable bond < 5) (Table 8). To be administered as drugs, all compounds must have no carcinogenic effect and pass the Lipinski filtering [44]. However, two chemical compounds, 3,4-dihydroxybenzoic acid and vanillic acid, showed positive response to blood-brain barrier, which indicates that all drugs will go through blood-brain barrier. These two compounds had weak inhibition of the human ether-a-go-go-related gene (hERG). Complete inhibition of hERG gene leads to long QT syndrome, so further study on this aspect is required [45]. P-glycoprotein (P-gp) functions as drug transporter, so clarification of the ligand noninhibitory activity towards P-gp is required. Both ligand molecules had noninhibitory activity towards P-gp, which confirms positive attributes of the ligand. Oral bioavailability confirms the efficacy of the compound.

### 3.13. Docking

In the present study, the following PubChem CIDs were denoted as D1, D2, and D3: 72[IUPAC name: 3,4-dihydroxybenzoic acid]; 8468[IUPAC name: 4-hydroxy-3-methoxybenzoic acid]; and 11228183 [IUPAC name: 3-[[2-[2-[2-[[(2S, 3R)-2-[[(2S, 3S, 4R)-4-[[(2S, 3R)-2-[[6-amino-2-[(1S)-3-amino-1-[[(2S)-2,3-diamino-3-oxopropyl]amino]-3-oxopropyl]-5-methylpyrimidine-4-carbonyl]amino]-3-[(2R, 3S, 4S, 5S, 6S)-3-[(2R, 3S, 4S, 5R, 6R)-4-carbamoyloxy-3,5-dihydroxy-6-(hydroxymethyl)oxan-2-yl]oxy-4,5-dihydroxy-6-(hydroxymethyl)oxan-2-yl]oxy-3-(1H-imidazol-5-yl)propanoyl]amino]-3-hydroxy-2-methylpentanoyl]amino]-3-hydroxybutanoyl]amino]ethyl]-1,3-thiazol-4-yl]-1,3-thiazole-4-carbonyl]amino]propyl-dimethylsulfanium]. On the other hand, PubChem CIDs 247, 305, 73170, and 73659 were excluded from docking study as they show carcinogenic properties in ADMET filtering process.

Total internal energy, total intramolecular energy, and torsional free energy minus energy of unbound system summed up the binding energy in docking study. In AutoDock Vina, based on the binding energy value, the top nine conformers were generated for each docked complex [37]. Among these conformers, the lowest binding energy was considered as the most favorable docking pose. The docking energy of ligand molecules and NF-κB protein in AutoDock Vina was D1: −7.2.kcal/mol, D2: −7.9 kcal/mol, and D3: −7.8 kcal/mol (Figure 6(a)); for Bcl-2 protein binding affinity was −7.9, −8.1, and −7.7 kcal/mol (Figure 6(b)), respectively, for D1, D2, and D3.

D1 compound showed binding energy of −7.2 kcal/mol by forming five hydrogen bonds at Asn103, His110, Ser211, Val212, and Arg214. Two hydrophobic bonds were also present at Val213 and Met208 for D1 and NF-*κ*B protein complex. On the other hand, D2 and NF-*κ*B complex were stabilized by hydrogen bond at Ser113, Arg157, and His112 position and hydrophobic bond at Leu143 and Val145 residue. Bleomycin compound formed multiple hydrogen bonds with NF-*κ*B protein at Ser249, Asn250, Asp274, Gly69, Lys80, Ser74, and Lys52 position and four hydrophobic bonds were seen at Gly55, Glu344, Lys79, and Lys80 residues.

**Figure 7:**
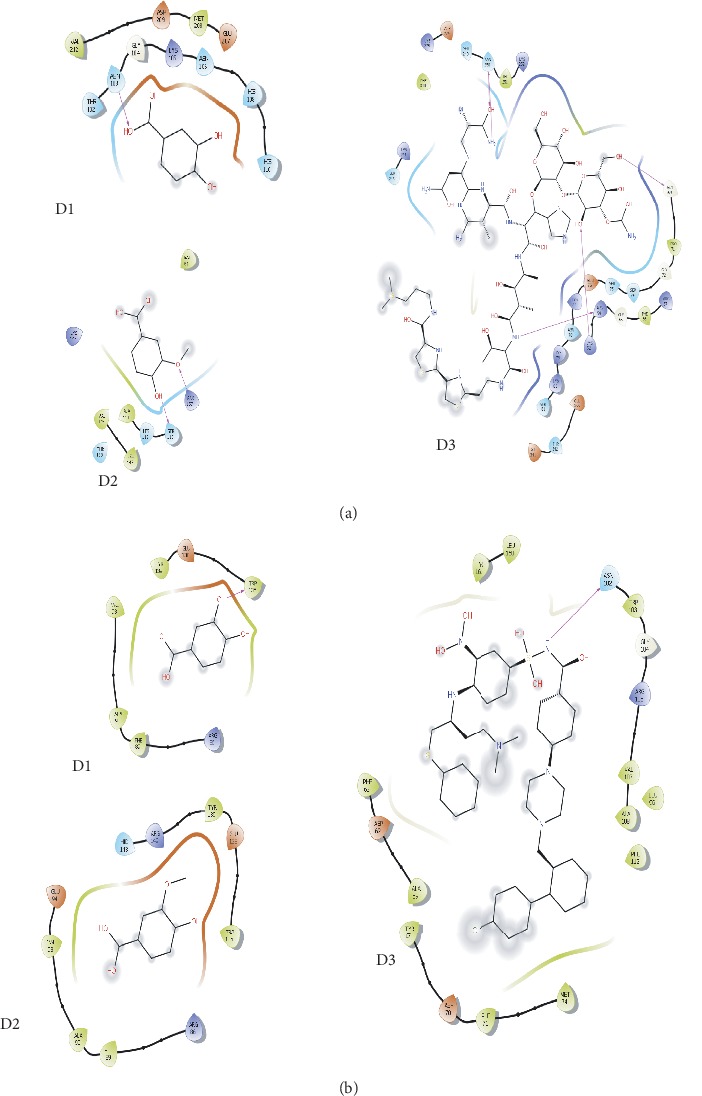
(a) Nonbonded interaction of NF-*κ*B with D1, D2, and D3. (b) Binding pose and interaction diagram of all Bcl-2 docked complexes.

On the other hand, three chemical compounds showed better energy while interacting with Bcl-2 protein. D1 and Bcl-2 complex had binding energy of −7.9 kcal/mol by forming three hydrogen bonds at Arg86, Trp135, and Glu138 and two hydrophobic bonds at Phe89 and Ala90. D2 compound also formed three hydrogen bonds with Bcl-2 protein at Ala90, Trp135, and Glu138 residue and two hydrophobic bonds were found at Phe89 and Ala90. However, D3 or bleomycin and protein complex were stabilized by three hydrogen bonds at Asn102, Gly104, Tyr161, and Leu160 and a hydrophobic bond at Phe63, Tyr67, Ala108, Ala59, Val107, Arg105, and Arg108 position (Figure7).

## 4. Discussion

Phytochemicals like alkaloids, glycosides, terpenoids, steroids, and flavonoids have anticancer activity. Comprehensive review has been done on the ability of antioxidant compounds to repel various human cancers [46]. Phenolics and flavonoids are the most abundant antioxidants found in the plant kingdom, and a number of plant species rich in phenolics and flavonoids reportedly have high therapeutic efficiency for the treatment of cancer [47]. In this investigation, we found that the bark extract of *A. augusta* is a rich source of phenolic and flavonoid compounds that can be used to treat various ailments.

The free radicals can initiate chain reactions and can cause cell death. Antioxidants accomplish these chain reactions by removing free radical intermediates and inhibit other oxidation reactions. Recent biological studies suggest that antioxidant rich plants can induce apoptosis of cancer cells in case of cancer (e.g., breast, liver, and colon) [48, 49]. Our result demonstrates that the methanolic bark extract of *A. augusta* has significant antioxidant activity with IC_50_ value of 38.65 *μ*g/ml when compared with standard BHT (IC_50_ value 57.29 *μ*g/ml).

In this study the cytotoxicity of the bark extract of *A. augusta* was reckoned against Ehrlich ascites carcinoma (EAC) cells at the concentration of 31.25–500 *μ*g/ml and showed promising cell growth inhibition. The IC_50_ value was calculated for the extract (329.41 *μ*g/ml) (Figure 2). To check whether the extract is too much toxic to the normal cells, the brine shrimp lethality bioassay was carried out. Probit analysis of the bioassay shows that the toxicity of the extract is comparatively low compared to that to the EAC cells.

*In vivo* anticancer activity of the bark extract was assessed by using EAC cell-bearing mouse model, and the result was compared with a reference standard drug named bleomycin (0.3 mg/kg). The numbers of viable cells were found to be decreased by around 75.5% at dose 100 mg/kg body weight (i.p.). In this study, untreated EAC cell-bearing mice gained rapid tumor growth, but the bark extract-treated EAC cell-bearing mice exhibited cell growth inhibition significantly.

Apoptosis is a cell suicidal mechanism that can be regulated by numerous cellular signaling pathway characterized morphologically by cell shrinkage, membrane blebbing, chromatin condensation, apoptotic body formation, etc. [28]. Treated and control EAC cells stained with DAPI dye under fluorescence microscope observation showed different morphological changes which positively indicate the apoptosis of EAC cells treated with bark extract. It was strongly believed that cleavage of the chromosomal DNA into small fragment is the hallmark of the apoptosis [50]. We obtained smeared and fragmented band in 1% agarose gel in case of treated EAC cells that may be responsible for the occurrence of apoptosis, which might have taken place due to the presence of a protein called DNA fragmentation factor (DFF) shown by Wang and colleagues [51]. This could be due to the chromatin condensation and nuclear fragmentation, so this observation strongly proves that the cytotoxicity of bark extract occurred via apoptosis of the cancer cells.

Various genes are involved in the control of cell cycle, and upregulation or downregulation of particular gene can help in regaining the control of cell cycle in cancer cells, which can lead them to programmed cell death and limit cancer growth. Difference in expression level of p53, Bax, Bcl-2, and NF-*κ*B in treated and control cells is shown in Figure 6. The results of our study effectively suggest that the bark extract promotes the expression of p53 gene and inhibits the expression of Bcl-2 gene. The activation of tumor suppressor protein typically demonstrated the apoptosis of EAC cells treated with *A. augusta* bark extract, while the downregulation of Bcl-2 gene increased the expression of Bax, which promotes cell death; however, when Bcl-2 dominates, the apoptotic pathway is inhibited and cells survive [24, 52]. Again the expression of NF-*κ*B was downregulated in case of bark extract-treated cells similar to the downregulation of Bcl-2 which is also known to have supportive role in case of inhibition of cellular apoptosis [53]. The apoptosis induced by the bark extract of *A. augusta* in EAC cells was associated with upregulation of p53 expression mediated by inactivation of NF-*κ*B and production of ROS. According to previous studies, the production of intracellular ROS activated the downstream production of p53, which is a mitochondria-mediated pathway and consequently leads to apoptotic cell death [54, 55].

The polypharmacological profile of chemical compounds with the ability to target multiple biological signaling systems could be beneficial for treating complex diseases. Absorption, blood-brain barrier penetration, and bile elimination of chemical compounds can depend on the MW profile of ligand molecules. On the other hand, the Log *p* value or lipophilicity profile of the ligand is pivotal for determining potency and several pharmacological parameters. Permeability can be decreased when lipophilicity value becomes too low, whereas metabolism is more likely to be compromised at high lipophilicity value. Therefore, carcinogenic profile and Lipinski need to be carefully assessed to declare the chemical compound as a lead molecule [56, 57]. Two compounds of *A. augusta* passed pharmacological filtering and can be targeted in the future for further research.

Ligand protein binding is thought to be facilitated by the hydrogen bonding pattern in a biological system. Although the contribution of hydrogen bond to molecular function is not well understood, the presence of hydrogen bond can significantly improve the binding energy of protein and ligand complex [58–60]. Lastly, we found that among three ligands (including bleomycin), D2 exhibited the most potent anticancer activity against Bcl-2 and NF-*κ*B by showing binding affinity (−7.9, −8.1). D2 or vanillic acid created strong noncovalent interaction at Ala90 which is near to the active sites of Bcl-2 protein (Val90, Glu95, Glu96). Based on the binding energy, this result suggests that D2 or vanillic acid may have the capacity to interfere with Bcl-2 and NF-*κ*B which ultimately can lead to further investigation of the *A. augusta* compound. Vanillic acid or D2 compound has been reported in the prevention of alternation of ion channels and cardiomyocyte death in mice, with antifungal, antimicrobial, and acetylcholinesterase inhibitory activities. Interestingly, derivatives of vanillic acid can act as immunosuppressive agents, and the molecular modeling profile was explored [61–63].

Bcl-2 can be overexpressed in various types of solid cancer and prevents apoptosis induced by a protective cell signaling mechanism. Furthermore, oligomerization prevention and apoptosis cascade initiation can be provided by the BH3 domain of Bcl-2 protein. The natural phenolic compound 3,4-dihydroxybenzoic acid or protocatechuic acid can be used as a protective agent against neoplasm and is mostly associated with antioxidant activity [62]. Protocatechuic acid or D1 has been recently reported to inhibit Bcl-2 protein expression and retinoblastoma phosphorylation in human leukemia cells [64]. Generally, NF-*κ*B translocates in the nucleus from cytoplasm by LPS (lipopolysaccharides) stimulation and, thus, transcription of inflammatory mediators induced by NF-*κ*B in the nucleus [64–69]. However, the impact of protocatechuic acid on LPS-induced NF-*κ*B suggests that PA can inhibit NF-*κ*B in BV2 microglia cells. Besides, LPS-induced TLR4 activation was suppressed by protocatechuic acid which is ultimately responsible for NF-*κ*B inhibition by D1 or protocatechuic acid [70].

This combinatorial approach will aid fellow researchers in isolating compounds from *Abroma augusta* and getting more view for desirable consideration in future investigation.

## 5. Conclusion

In this study, we tried to provide fruitful evidence supporting the toxicity of *A. augusta* to EAC cells. Again, the results derived from this study strongly proves that *A. augusta* bark extract can induce EAC cells apoptosis through the activation of p53, Bax and also the suppression of NF-*κ*B, Bcl-2. The apoptotic effect of methanolic extract against EAC cells is attributed to its antioxidant activity and high phenolic and flavonoid contents. Further, activity of the chemical compound found in *A. augusta* through docking simulation has shown positive effect against Bcl-2 and NF-*κ*B. However, in vitro and in vivo studies on the probable active compounds of this plant responsible for the above activities are still required to be investigated.

## Figures and Tables

**Table 1:** The primer used for PCR amplification.

Gene	Primer sequence	Amplification size
GAPDH	Forward: 5′-GTGGAAGGACTCATGACCACAG-3′ Reverse: 5′-CTGGTGCTCAGTGTAGCCCAG-3′	350 bp
p53	Forward: 5′-GCGTCTTAGAGACAGTTGCCT-3′ Reverse: 5′-GGATAGGTCGGCGGTTCATGC-3′	450 bp
Bax	Forward: 5′-GGCCCACCAGCTCTGAGCAGA-3′ Reverse: 5′-GCCACGTGGGCGTCCCAAAGT-3′	500 bp
Bcl-2	Forward: 5′-GTGGAGGAGCTCTTCAGGGA-3′ Reverse: 5′-AGGCACCCAGGGTGATGCAA-3′	250 bp
NF-*κ*B	Forward: 5′-AACAAAATGCCCCACGGTTA-3′ Reverse: 5′-GGGACGATGCAATGGACTGT-3′	125 bp

**Table 2:** Binding energy, hydrogen bond, hydrophobic interaction, and binding distance of docked pose of NF-*κ*B, with kcal/mol used as unit of binding affinity

Compound	Docking score (kcal/mol) in AutoDock	Docking score in MOE	H-bond interactions	Hydrophobic interactions
			Amino acids distance (Å)	Amino acids distance (Å)
D1	−7.2	−11.28	Asn103 (2.08), His110 (2.09), Ser211 (2.29), Val212 (2.55), Arg214 (2.56)	Val213 (2.86), Met208 (3.56)
D2	−7.9	−11.75	Ser113 (2.40), Arg157 (2.05), His112 (3.02)	Leu143 (4.52), Val145 (4.79)
D3 or bleomycin	−7.8	−10.78	Ser249 (2.74), Asn250 (2.02), Asp274 (2.72), Gly69 (2.80), Lys80 (2.73), Ser74 (2.31), sLys52 (2.25)	Gly55 (3.73), Glu344 (3.21), Lys79 (4.45), Lys80 (3.28)

**Table 3:** Binding energy, hydrogen bond, hydrophobic interaction, and binding distance of docked pose of Bcl-2, with kcal/mol used as unit of binding affinity.

Compound	Docking score (kcal/mol) in AutoDock	Docking score in MOE	H-bond interactions	Hydrophobic interactions
			Amino acids distance (Å)	Amino acids distance (Å)
D1	−7.9	−11.29	Arg86 (2.3 Å), Trp135 (1.9 Å), Glu138 (2.2 Å)	Phe89 (5.1 Å), Ala90 (5.1 Å)
D2	−8.1	−11.26	Ala90 (2.9 Å), Glu138 (2.4 Å), Trp135 (2.5)	Phe89 (5.1 Å), Ala90 (5.1 Å)
D3	−7.7	−10.75	Asn102 (2.40), Gly104 (2.59), Tyr161 (2.05), Leu160 (2.40)	Phe63 (3.23), Tyr67 (5.15), Ala108 (5.02), Ala59 (4.47), Val107 (5.13), Arg105 (4.23), Ala108 (3.41)

**Table 4:** Results of phytochemical screening of *A. augusta*.

Phytochemicals	Tests	*A. augusta*
Alkaloids	Mayer's test	+
Saponins	Forth test	−
Glycosides	Keller–Kiliani test	+
Reducing sugars	Fehling test	+
Terpenoids	Salkowski's test	+
Steroids	Liebermann–Burchard test	+
Tannins	Ferric chloride test	−
Flavonoids	Ethyl acetate test	+

**Table 5:** Polyphenol contents of the methanolic bark extract of *A. augusta*.

Extract	Phenolics (GAE/g of dry extract)	Flavonoids (CAE/g of dry extract)
*A. augusta* (bark)	182.33 ± 3.055	81.33 ± 2.081

Data are represented as mean ± SD (*n* = 3).

**Table 6:** Hemolytic assay of bark extract of *A. augusta*.

Concentration (*μ*g/ml)	Absorbance at 540 nm	% hemolysis
Negative control	0.045	0
Positive control	0.478	95.35
250	0.045	9.41
500	0.020	4.2
1000	0.0196	4.1

**Table 7:** Effect of bark extract of *A. augusta* on EAC cell growth in mice (*in vivo*).

Name of experiment	Dose (mg/kg b. wt./day, i.p.)	Viable EAC cells on day 6 after inoculation	% of cell growth inhibition
EAC + control	—	2.675 ± 0.1890 × 10^6^	—
EAC + bleomycin	0.3	0.423 ± 0.0516 × 10^6^	84.143
EAC + *A. augusta*	50	1.21 ± 0.1897 × 10^6^	54.9
EAC + *A. augusta*	100	0.65 ± 0.0894 × 10^6^	75.5

**Table 8:** Predicted pharmacological and pharmacokinetics properties of selected *A. augusta* chemical compounds; here only two compounds passed the Lipinski filtering and toxicity scale.

Parameter	CID-72	CID-247	CID-305	CID-8468	CID-73170	CID-73659
Blood-brain barrier	0.6376 (−)	0.9463 (+)	0.9214 (+)	0.5146 (−)	0.9618 (+)	0.6673 (+)
Human intestinal absorption	0.8811 (+)	0.8819 (+)	0.9640 (−)	0.9231 (+)	1 (+)	0.9770 (+)
P-gp inhibitor	0.9948 Noninhibitor	0.9694 Noninhibitor	0.9540 Noninhibitor	0.9165 Noninhibitor	0.7670 Noninhibitor	0.8907 Noninhibitor
Log *p*	0.65	−1.69	−1.38	1.08	7.05	5.24
hERG Inhibitor	0.9508 Noninhibitor	0.9340 Noninhibitor	0.8071 Noninhibitor	0.9545 Noninhibitor	0.7513 Noninhibitor	0.7017 Noninhibitor
AMES toxicity	0.9326 Non AMES toxic	0.9331 Non AMES toxic	0.9386 Non AMES toxic	0.9419 Non AMES toxic	0.8996 Nontoxic	0.9087 AMES toxic
Carcinogen	0.9154 Noncarcinogens	0.7504 Carcinogens	0.5996 Carcinogens	0.9046 Noncarcinogens	0.9227 Noncarcinogens	0.9552 Noncarcinogen
Molecular weight	154.12	117.15	104.17	168.15	426.73	472.70
Num. rotatable bonds	1	2	2	2	0	1
Hydrogen bond acceptors	4	2	1	4	1	4
Hydrogen bond donors	3	0	1	2	1	3
Molar refractivity	37.45	28.35	29.69	41.92	135.14	137.82
TPSA	77.76	40.13	20.23	66.76	20.23	77.76
Lipinski	Yes	No (1 violation)	Yes	Yes	No (1 violation)	No (1 violation)
Bioavailability	0.56	0.55	0.55	0.55	0.55	0.56

## Data Availability

The data used to support the findings of this study are available from the corresponding author upon request.

## Supplementary Materials

Supplementary Materials Supplementary Table S1: the mortality percentage of brine shrimp nauplii induced by methanolic bark extract of *A. augusta*. Supplementary Figure S2: the percentage of mortality of bark extract against different concentrations.
