# Supplementary material for: Methanolic Bark Extract of Abroma augusta (L.) Induces Apoptosis in EAC Cells through Altered Expression of Apoptosis Regulatory Genes
Source: Evid Based Complement Alternat Med. 2020 Apr 13;2020:9145626. doi: 10.1155/2020/9145626 (PMC7178513; doi:10.1155/2020/9145626)
Supplement: Supplementary Materials — Supplementary Table S1: the mortality percentage of brine shrimp nauplii induced by methanolic bark extract of A. augusta. Supplementary Figure S2: the percentage of mortality of bark extract against different concentrations. [file 9145626.f1.docx]

**Supplementary Materials**

**Supplementary Table S1: The mortality % of Brine Shrimp nauplii induced by methanolic bark extract of *A. augusta***

| **Dose (µg/ml)** | **Log dose** | **Number of nauplii** | **Kill** | **% Kill** | **% Cr** | **E. Pr** | **Ex. Pr** | **Wk. Por** | **Weight** | **P. Pro** |
| --- | --- | --- | --- | --- | --- | --- | --- | --- | --- | --- |
| 25 | 1.397926 | 10 | 1 | 10 | 10 | 3.72 | 3.565591 | 3.75 | 2.69 | 3.5396 |
| 50 | 1.698952 | 10 | 1 | 10 | 10 | 3.72 | 3.919227 | 3.74 | 4.05 | 3.907529 |
| 75 | 1.875042 | 10 | 2 | 20 | 20 | 4.16 | 4.126091 | 4.17 | 4.71 | 4.122754 |
| 100 | 1.999979 | 10 | 2 | 20 | 20 | 4.16 | 4.272864 | 4.15 | 5.03 | 4.275458 |
| 150 | 2.176069 | 10 | 3 | 30 | 30 | 4.48 | 4.479728 | 4.48 | 5.58 | 4.490683 |
| 200 | 2.301006 | 10 | 4 | 40 | 40 | 4.75 | 4.6265 | 4.74 | 6.01 | 4.643387 |

Y = 1.830992 + 1.222246 X

Chi-squared is 0.379103 with 4 degree of freedom

LOG LD_50_ is 2.592775

LD_50_ is 391.539

%Cr=percentage Correlation; E.Pr=Empirical probit; Ex. Pr=Experimental Probit; WK.pro=Work probit; P.pro=Final probit

**Supplementary Figure S2:** % of mortality of bark extract against different concentrations
